# Supplementary material for: A comparison of approximate versus exact techniques for Bayesian parameter inference in nonlinear ordinary differential equation models
Source: R Soc Open Sci. 2020 Mar 11;7(3):191315. doi: 10.1098/rsos.191315 (PMC7137938; doi:10.1098/rsos.191315)
Supplement: ESM descriptions and Information in How to run the R code [file rsos191315supp1.txt]

R code for producing the results appeared in the paper 1- The descriptions for the files:R code for example 1 in the paper:1)Run_file_final.R : The main R code that Run all the methods.2)Initial_parameters_final.R : File generate all the initial value for the parameters in example 1.3) ABC_MCMC_SIR_final.R: File contains all the function needed to implement MCMC, ABC SMC, ABC SMC with adaptive distance and function to implement Vaart’s method. 4) Sir_data.rds: Sir data that used on example 1.5)Fig_2.R: R code that produce the figure number 2 in first example.6)Fig_3.R: R code that produce the figure number 3 in first example.7) Fig_4.R: R code that produce the figure number 4 in first example.8)Fig_5: R code that produce the figure number 5 in first example.9) Fig_6.R: R code that produce the figure number 6 in first example.10) Fig_7.R : R code that produce the figure number 7 in first example. R code for example 2 in the paper:11) Run_file_Malariah.R: The main R code that Run all the methods.12) Initial_parameters_Malariah_final.R : File generate all the initial value for all the parameters.13) ABC_MCMC_Malariah.R: File contains all the function needed to implement MCMC, ABC SMC and ABC MCMC.14) Malariah_data.rds : Malariah Data used on example 2.15) Fig_9.R: R code that produce the figure number 9 in Second example. 16) Fig_10.R: R code that produce the figure number 10 in Second example. 2- Information in How to run the R code for Example 1 and 2:#######Code and data to generate results for The First Example:To run the software please Run the main file “Run_file_final.R ”(File that Run all the methods) which will call the data, functions and the initial values from “Sir_data.rds”, “ABC_MCMC_SIR_final.R” and “Initial_parameters_final.R”.#######Code and data to generate results for Second Example:To run the software please Run the main file “Run_file_Malariah.R” (File that Run all the methods.) which will call the data, functions and the initial values from “Malariah_data.rds ” , “ABC_MCMC_Malariah.R:” and “ Initial_parameters_Malariah_final.R”.
