# Supplementary material for: A comparison of approximate versus exact techniques for Bayesian parameter inference in nonlinear ordinary differential equation models
Source: R Soc Open Sci. 2020 Mar 11;7(3):191315. doi: 10.1098/rsos.191315 (PMC7137938; doi:10.1098/rsos.191315)
Supplement: Electronic supplementary material discretion file [file rsos191315supp2.rtf]

Electronic supplementary material discretion file:#######Code and data to generate results for The First Example:To run the software please Run the main file “Run_file_final.R ”(File that Run all the methods) which will call the data, functions and the initial values from “Sir_data.rds”, “ABC_MCMC_SIR_final.R” and “Initial_parameters_final.R”.The description for each file as below:  1)Run_file_final.R : File that Run all the methods.2)Initial_parameters_final.R : File generate all the initial value for the parameters.3) ABC_MCMC_SIR_final.R: File contains all the function needed to implement MCMC, ABC SMC, ABC SMC with adaptive distance and function to implement Vaart’s method. 4) Sir_data.rds: Sir data that used on example 1.####### Fig_2.R, Fig_3.R, Fig_4.R, Fig_5.R, Fig_6.R, to Fig_7 : files that produce the figures for First example #######Code and data to generate results for Second Example:To run the software please Run the main file “Run_file_Malariah.R” (File that Run all the methods.) which will call the data, functions and the initial values from “Malariah_data.rds ” , “ABC_MCMC_Malariah.R:” and “ Initial_parameters_Malariah_final.R”.The description for each file as below:  1) Run_file_Malariah.R: File that Run all the methods.2) Initial_parameters_Malariah_final.R : File generate all the initial value for all the parameters.3) ABC_MCMC_Malariah.R: File contains all the function needed to implement MCMC, ABC SMC and ABC MCMC.4) Malariah_data.rds : Malariah Data used on example 2.####### Fig_9.R, Fig_10.R: files that produce the figures for Second Example. 
