# Supplementary material for: A comparison of approximate versus exact techniques for Bayesian parameter inference in nonlinear ordinary differential equation models
Source: R Soc Open Sci. 2020 Mar 11;7(3):191315. doi: 10.1098/rsos.191315 (PMC7137938; doi:10.1098/rsos.191315)
Supplement: Electronic supplementary material discretion file [file rsos191315supp3.rtf]

Electronic supplementary material discretion file:#Code and data to generate results for example1:1)Initial_parameters_final.R : File generate all the initial value for the parameters.2) ABC_MCMC_SIR_final.R: File contains all the function needed to implement MCMC, ABC SMC, ABC SMC with adaptive distance and function to implement Vaart’s method. 3) Run_file_final.R: File that Run all the methods.4) Sir_data.rds: Sir data that used on example 2.#Code and data to generate results for example2: 1) Initial_parameters_Malariah_final.R : File generate all the initial value for all the parameters.2) ABC_MCMC_Malariah.R: File contains all the function needed to implement MCMC, ABC SMC and ABC MCMC.3) Run_file_Malariah.R: File that Run all the methods.4) Malariah_data.rds : Malariah Data used on example 2.# Fig_2 to Fig_10 : files that produce the figures on the paper.
